# Synthesis, characterization, and application of 2D/2D TiO_2_-GO-ZnFe_2_O_4_ obtained by the fluorine-free lyophilization method for solar light-driven photocatalytic degradation of ibuprofen

**DOI:** 10.1007/s11356-022-24587-0

**Published:** 2022-12-20

**Authors:** Izabela Malinowska, Paweł Kubica, Piotr Madajski, Adam Ostrowski, Cristina Gómez Polo, Laura Carvera, Waldemar Bednarski, Anna Zielińska-Jurek

**Affiliations:** 1 Department of Process Engineering and Chemical Technology, Gdansk University of Technology, Narutowicza 11/12, 80-233 Gdansk, Poland; 2 Department of Analytical Chemistry, Gdansk University of Technology, Gdansk, Poland; 3 Faculty of Chemistry, Nicolaus Copernicus University, Gagarina 7, 87-100, Toruń, Poland; 4 Institute of Molecular Physics, Polish Academy of Sciences, Smoluchowskiego 17, 60-179 Poznań, Poland; 5 Departamento de Ciencias-INAMAT2, Universidad Pública de Navarra, Campus de Arrosadia, 31006 Pamplona, Spain

**Keywords:** Magnetic photocatalyst, Magnetic separation, Ibuprofen degradation, TiO_2_ nanosheets, Pharmaceuticals degradation, Photocatalysis

## Abstract

In this study, we report the potential of 2D/2D TiO_2_-GO-ZnFe_2_O_4_ photocatalyst obtained using the fluorine-free lyophilization technique for the degradation of ibuprofen belonging to the group of active pharmaceutical ingredients (API). The improved ibuprofen degradation under simulated solar light was achieved in the presence of a composite of 2D TiO_2_ combined with GO and embedded ZnFe_2_O_4_, which additionally provides superparamagnetic properties and enables photocatalyst separation after the photodegradation process. After only 20 min of the photodegradation process in the presence of 2D/2D TiO_2_-GO-ZnFe_2_O_4_ composite, more than 90% of ibuprofen was degraded under simulated solar light, leading to non-toxic and more susceptible to biodegradation intermediates. At the same time, photolysis of ibuprofen led to the formation of more toxic intermediates. Furthermore, based on the photocatalytic degradation analysis, the degradation by-products and possible photodegradation pathways of ibuprofen were investigated. The photodegradation tests and electronic spin resonance analyses indicated the significant involvement of superoxide radicals and singlet oxygen in the ibuprofen photodegradation process.

## Introduction

Ibuprofen (IBU), i.e., isobutylphenylopropionic acid, is the third most commonly used worldwide over-the-counter, non-steroidal anti-inflammatory drug to treat pain of various origins. IBU world production is several kilotons per year, and due to the increased consumption rate, it has been detected at the level from hundreds of ng·dm^−3^ to a few μg·dm^−3^ in surface waters and wastewater treatment effluents (Buser et al. 1999; Lee et al. 2003).

In 2003, Andreozzi et al. (2013) noticed the presence of ibuprofen and other emerging contaminants in European cities river waters, including France, Italy, Sweden, and Greece. Ibuprofen was detected in a wide concentration range of 0.05 μg·dm^−3^ to 7.11 μg·dm^−3^. In 2007, ibuprofen was found in wastewater from northern Scotland at a concentration of 0.405 μg·dm^−3^ (Nebot et al. 2007). The reported results indicate that the scale of the problem is local and global.

However, the presence of trace amounts of pharmaceuticals is not so dangerous as their ability to bioaccumulate and biomagnify in living organisms. The detrimental effect depends on the cellular level (Wang et al. 2016). In the case of fish, IBU impairs the reproductive system (Flippin et al. 2007). For example, Gagne et al. studied the effect of IBU on rainbow trout and observed an enzymatic and metabolite disorder in this group of organisms (Gagne et al. 2006). Hayashi et al. (2008) studied the effect of the drug on crustaceans Daphnia magna. Therefore, it is essential to prevent ibuprofen and other emerging contaminants from being released into the environment by applying advanced water treatment technologies complementary to commonly used biological degradation processes.

In the group of advanced oxidation processes, photocatalysis has been proposed as one of the green technologies for degradation of emerging contaminants. Photocatalysis is an artificial photosynthesis technique, which can be conveniently employed as a tertiary treatment process in existing wastewater treatment plants. Chemically stable semi-conductors can produce reactive oxygen species upon irradiation and degrade xenobiotics into non-toxic compounds (Li et al. 2020). However, their photocatalytic activity is usually limited as sunlight-driven photocatalysts due to large bandgap and relatively low quantum yield efficiency resulting from charge carrier recombination.

In 2D nanostructures, the single-crystalline characteristic allows fast in-plane electron transport (Sheng et al. 2017; Nguyen-Phan et al. 2011). Graphene oxide possesses great adsorption capacity for organic compounds through strong π-π interactions. Therefore, to further enhance the performance of TiO_2_, combining titanium (IV) oxide with 2D graphene oxide as a co-catalyst is an effective approach to improve the surface adsorption of organic pollutants and inhibit electron–hole recombination (Nguyen-Phan et al. 2011). Recently, Erim and co-workers applied 3D TiO_2_/2D GO/chitosan photocatalyst for the photodegradation of cefixime, an antibiotic used against gram-negative and gram-positive aerobic bacteria infections. The efficiency of TiO_2_ supported by graphene oxide cross-linked with chitosan reached 95% in 60 min of photodegradation in the presence of UVA light (Erim et al. 2021).

Recently, in various efforts to extend TiO_2_ photocatalytic activity, special attention has been focused on TiO_2_ morphology and microstructure design to enhance emerging contaminant photodegradation. Although visible-light active titanium (IV) oxide photocatalysts require chemical modification, their total efficiency has significantly improved by controlling the photocatalyst morphology (Dudziak et al. 2021; Sajan et al. 2016). In recent years, two-dimensional TiO_2_ nanomaterials have attracted considerable attention due to developed specific surface area and structural coherence in the width dimension.

Furthermore, hybridization of semi-conductor material with magnetic particles enables to overcome of another challenge in photocatalysis related to the separation of photocatalyst nanoparticles from post-process suspension. Graphene oxide and reduced graphene oxide can form stable bonds with TiO_2_ and ferrite particles incorporating magnetic properties that facilitate the separation of photocatalyst after wastewater treatment. Aflatoxin B_1_, one of the most toxic among the mycotoxins, was successfully degraded to less harmful by-products in the presence of Fe_3_O_4_/GO/3D TiO_2_ photocatalyst under UV–vis light (Suna et al. 2021).

In this regard, in the present work, a new 2D/2D TiO_2_-GO-ZnFe_2_O_4_ composite was obtained and for the first time used for ibuprofen photocatalytic degradation under simulated solar light. Regarding sustainable development and green chemistry, 2D TiO_2_ photocatalyst structures with exposed {0 0 1} facets were synthesized using the fluorine-free lyophilization technique as a green concept for the synthesis. The as-obtained 2D anatase nanosheets were used as building blocks for hybridization with graphene oxide to enhance the photocatalyst performance by (1) maximization of UV–vis light absorption, (2) increasing the lifetime of photoexcited electrons and holes, (3) improvement of transport kinetics to surface reaction sites, and (4) increasing of the reaction kinetics on the surface sites. Furthermore, incorporating zinc ferrite particles with superparamagnetic properties allowed for the separation of 2D/2D TiO_2_-GO-ZnFe_2_O_4_ ternary composite material in an external magnetic field. The reactive oxygen species participating in the photocatalytic degradation were investigated by reference experiments in the presence of scavengers. Based on LC–MS analysis, intermediates were determined in the photocatalytic reaction, and a possible mechanism of ibuprofen photodegradation was proposed. Moreover, the toxicity of ibuprofen and its photodegradation intermediates was studied to specify whether the degradation process in the presence of 2D/2D TiO_2_-GO-ZnFe_2_O_4_ can reduce the acute toxicity of active pharmaceutical ingredients.

## Experimental

### Materials

Aqueous ammonia (25%), hydrogen peroxide (30%), and titanium(IV) oxysulphate were provided by Sigma Aldrich and used for titanium(IV) oxide synthesis. Graphene oxide, zinc chloride, iron sulfate heptahydrate, and sodium hydroxide were purchased from Aldrich (Germany). POCH Gliwice (Poland) supplied cationic surfactant hexadecyltrimethylammonium bromide (CTAB). Benzoquinone (reagent grade, ≥ 98%) and *tert*-butanol (anhydrous, ≥ 99.5%) were purchased from Sigma Aldrich, POCH Gliwice provided AgNO_3_ (pure p.a.) and EDTA (pure p.a.).

### Preparation of 2D anatase nanosheets

First, 7.6 g of TiOSO_4_ was dissolved in 100 cm^3^ of deionized water (DI-H_2_O), heated up to 35 °C until clarification of the solution, and then cooled to 0 °C using an ice bath. Then, ammonia solution (25%) was added until the pH of 8. The obtained precipitate was washed several times with deionized water, centrifuged and suspended in DI-H_2_O. Furthermore, hydrogen peroxide solution (45%) was added, which caused a decrease in pH from 8 to 2 and changed the suspension color from white to yellow. The suspension was stirred for 30 min at room temperature until a clear solution formed. The solution was aged for 48 h at 3 °C to form a yellow gel that was further lyophilized at -84 °C and a pressure of 0.3 Pa. The lyophilized material was calcined at 700 °C for 1 h with the formation of TiO_2_ nanosheets.

### Preparation of 2D/2D TiO_2_-GO

Graphene oxide (0.03 g) was dispersed in 50 cm^3^ of ethanol (EtOH) and deionized water (70:30) solution and ultrasonicated for 30 min to isolate graphite layers. Subsequently, 3 g of 2D TiO_2_ (prepared according to the procedure given above) was added and stirred for 6 h, placed in a stainless steel autoclave lined with Teflon, and subjected to hydrothermal reaction at 130 °C for 6 h. The amount of graphene oxide relative to TiO_2_ was 1%. The final product 2D/2D TiO_2_-GO was separated, purified with a mixture of EtOH/DI-H_2_O (1:1) several times, and dried to dry mass at 70 °C.

### Preparation of 2D/2D TiO_2_-GO-ZnFe_2_O_4_ photocatalysts

First, to obtain zinc ferrite particles, FeSO_4_∙7H_2_O and ZnCl_2_ were dissolved in a stoichiometric 2:1 (Fe: Zn) molar ratio in deionized water under stirring (500 rpm) for 30 min. Afterwards, the metals were precipitated from the homogenous solution by adding 5 M NaOH at room temperature until the pH was 12. The suspension was transferred into an autoclave and treated at 200 °C for 5 h. Subsequently, the obtained magnetic particles were separated in the magnetic field, washed several times with deionized water, and dried at 100 °C to dry mass.

Furthermore, ZnFe_2_O_4_ nanoparticles (0.1 g) were dispersed in 60 cm^3^ of 0.2 M CTAB in deionized water and ultrasonicated for 2 min. Then 20 cm^3^ of ammonia solution was added. The mixture was stirred for an hour, and 2 g of 2D TiO_2_-GO was added, mixed using a mechanical stirrer for 2 h and solvothermal treated in an autoclave at 30 °C for 24 h. The content of zinc spinel ferrite to TiO_2_-GO was 5% by weight. The obtained composite material was separated and washed with deionized water several times and dried at 70 °C. Finally, the obtained composite was calcined at 550 °C for 3 h.

### Characterization of 2D/2D TiO_2_-GO-ZnFe_2_O_4_photocatalysts

X-ray powder diffraction (XRD) analysis was performed using Rigaku MiniFlex 600.

X-ray diffractometer (Rigaku Corporation, Tokyo, Japan). The patterns were obtained in the range from 10° to 80° 2θ with a ∆2θ = 0.01°. The diffuse reflectance (DRS) spectra were analyzed using a Thermo Scientific Evolution 220 spectrophotometer (Waltham, MA, USA) with an integrating sphere. Brunauer–Emmett–Teller (BET) surface area analysis was performed using the Micromeritics Gemini V instrument. The morphology of the prepared photocatalysts was determined using Cs-corrected STEM (High Angle Annular Dark Field, HAADF) and HR-TEM (FEI Europe, Tencai F20 X-Twin) microscopy. For TEM analysis, 2D/2D TiO_2_-GO-ZnFe_2_O_4_ particles were dispersed in ethanol and placed in an ultrasound bath for 1 min. Subsequently, a few drops of suspension were deposited on the copper microgrid. Electrochemical analysis was performed using the potentiostat–galvanostat (AutoLab PGStat 302 N system, Utrecht, Netherlands) under GPS/FRA software control. Ag/AgCl (3 M KCl) was applied as a reference electrode, while platinum mesh was used as a counter electrode. The spectra were run at the frequency range from 20 kHz to 1 Hz with a 50 mV amplitude of the alternating current. In order to prepare the Mott–Schottky analysis, the fabricated titania powders were used to form the paste deposited using the doctor-blade technique onto the fluorine tin-doped oxide (FTO) support. The paste consists of deionized water, PEG (polyethylene glycol), Triton X-100, and the photocatalyst. Finally, the calcination was performed at 400 °C for 5 h with a heating rate of 1 °C·min^−1^, ensuring the removal of the organic binder. The fabricated electrode material stayed as a working electrode tested in a three-electrode arrangement, where Ag/AgCl/0.1 M KCl and Pt mesh were used as reference and counter electrodes, respectively. The deaerated 0.5 M Na_2_SO_4_ was used as an electrolyte. The electrochemical impedance spectroscopy (EIS) data was recorded from the anodic toward the cathodic direction using Autolab PGStat302N. EIS data were recorded in the potential range from + 1.0 to − 1.0 V vs. Ag/AgCl/0.1 M KCl for the frequency of 1000 Hz using a 10 mV amplitude of the AC signal. The capacitance of the space charge layer was further calculated from the imaginary part of the measured impedance following the equation: C SC = -1/2πfZ", where f stands for the frequency of the AC signal and Zim for the imaginary part of the impedance.

Electron spin resonance (ESR/EPR) spectra were recorded at room temperature using a BRUKER ELEX-SYS spectrometer (X-band) equipped with a highly sensitive ER4122 SHQE-W1 resonator. The samples were prepared as an aqueous suspension of 2DTiO_2_/GO/ZnFe_2_O_4_ (concentration 1.5 mg∙cm^−3^) in the presence of α-phenyl-N-tert-butylnitrone (PBN) spin trap (concentration 40 mmol·dm^−3^). Approximately 0.015 cm^3^ mixture of PBN and 2DTiO_2_/GO/ZnFe_2_O_4_ aqueous suspension was placed into a thin-walled quartz tube with 0.8 mm ID and put in an ESR cavity. During the EPR photochemical experiments performed under aerobic and hypoxic conditions, the samples were irradiated directly in the EPR resonator with a light flux of 70 mW/cm^2^. The aerobic or hypoxic conditions were obtained by passing air or gaseous N_2_ through the samples prepared as suspensions. Magnetic measurements were performed with a SQUID magnetometer (Quantum Design MPMS XL7), enabling the characterization of the temperature dependence (5 – 300 K) of the magnetisation and the hysteresis loops of the samples.

### Photocatalytic degradation of ibuprofen

The photocatalytic activity was studied in the reaction of ibuprofen degradation. In this regard, 0.05 g of the photocatalyst particles was added to a quartz reactor containing 25 cm^3^ of ibuprofen (IBP) solution with an initial concentration of 21 mg·dm^−3^. The suspension was stirred and aerated. The reactor was irradiated using a 300 W Xenon lamp (LSH302, LOT-Quantum Design, Darmstadt, Germany) as ultraviolet–visible (UV–vis) light source. During the photocatalytic process, samples of 1 cm^3^ of the suspension were collected at regular time intervals. The ibuprofen degradation rate was measured as a pharmaceutical concentration decrease using liquid chromatography-quadrupole time of flight mass spectrometry (LC-TOFMS). Measurements were performed using LC system 1200 Infinity (Agilent, USA) with DAD (diode array detector) and QTOF 6540 (Agilent, USA). All modules were controlled by MassHunter v B9.0 and B7.0, and this software was used for data collection and processing. The final optimized method for the separation of possible by-products utilized the Zorbax XDB-C8 column (150 × 4.6 mm, 3 μm). The chromatographic conditions were as follows: flow rate 0.8 cm^3^·min^−1^ in isocratic mode consisting of 60% H_2_O and 40% of acetonitrile v/v, the separation temperature was kept at 35 °C, while injection volume was 0.005 cm^3^ in each analysis. The parameters of detection and ionization were as follows: data gathering in SCAN mode of MS in the range 50–300 m/z gas temperature 300 °C, drying gas flow 8 dm^3^·min^−1^, nebulizer gas flow 35 psig, the voltage of capillary, fragmentor, and skimmer were kept at 3500, 60, and 60 V, respectively.

### Toxicity assessment

The toxicity of the initial IBU solution and its by-products during degradation were based on the inhibition of the bioluminescence of marine bacteria *Vibrio fischeri* (Microtox Acute Reagent, Modern Water, UK). The toxicity tests were conducted with the standard Microtox Acute Toxicity Test Screening 81.9% protocol, and the luminescence was determined using Microtox Model 500 (Modern Water, UK). According to the formula below, the luminescence inhibition (LI%) of reconstituted bacteria in ibuprofen and its degradation products solution was calculated compared to a blank control.

1 $$LI\%=\frac{{LI}_{blank}-{LI}_{x}}{{LI}_{blank}}\bullet 100\%$$

The EC_50_ and TU_50_ values for the initial ibuprofen solution and its degradation products after 120 min of photolytic and photocatalytic processes were determined by running the standard Microtox Acute Toxicity Test Basic 81.9% protocol. EC_50_ value indicates the pollutant concentration causing a 50% reduction of the initial bacteria luminescence, thus meaning a lethal effect on 50% bacteria population. The toxic unit for the pharmaceutical solution was calculated as the reversal of the EC_50_ value multiplied by 100. The obtained toxicity data were recorded after 15 min of reconstituted bacteria solution exposure to each sample (before, during, and after photocatalytic degradation).

## Results

### Characterization of 2D/2D TiO_2_-GO-ZnFe_2_O_4_photocatalysts

The physicochemical characteristics of the magnetic photocatalysts, e.g., crystallite sizes, indirect band gap values, and BET surface areas, are given in Table 1.

**Table 1:** The physicochemical characteristics of 2D TiO_2_, 2D TiO_2_-GO, ZnFe_2_O_4_, and 2D/2D TiO_2_-GO-ZnFe_2_O_4_

Sample	BET (m^2^·g^−1^)	Pore volume (cm^3^/g)	Eg	Crystallite size (nm)		
				Anatase	Rutile	Zinc ferrite
2D TiO_2_	11.5	0.005	3.2	22	-	-
2D TiO_2_-GO	24.5	0.012	2.9	23	-	-
ZnFe_2_O_4_	21.5	0.002	1.5	-	-	9
2D TiO_2_-GO -ZnFe_2_O_4_	13.9	0.007	2.85	23	-	31

**Fig. 1:**
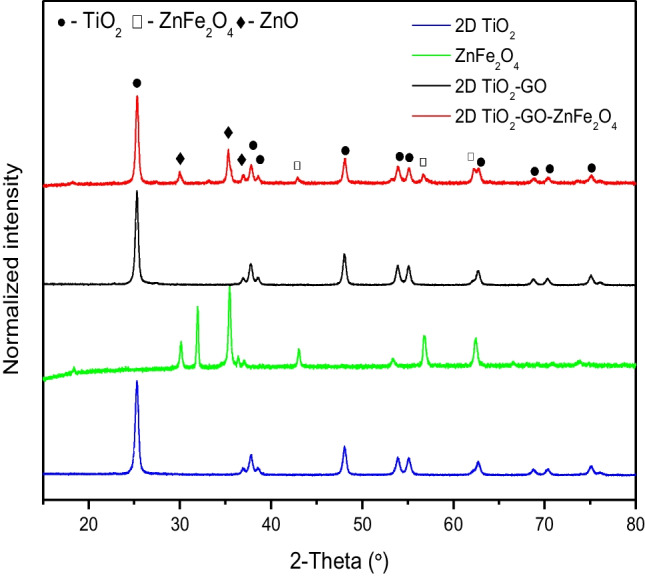
XRD diffraction patterns of the ZnFe_2_O_4_, 2D TiO_2_, 2D TiO_2_-GO, and 2D/2D TiO_2_-GO-ZnFe_2_O_4_ magnetic photocatalysts

The crystalline phase composition was examined by XRD analysis for both as-obtained zinc ferrite particles and magnetic ternary composites, and the results are presented in Fig. 1. The formation of ZnFe_2_O_4_ particles was confirmed by the presence of the signals at 2θ of 29.92°, 35.28°, 36.92°, 42.88°, 53.16°, 56.72°, and 62.24° corresponding to (220), (311), (222), (400), (422), (511), and (440) planes assigned to the cubic phase of the spinel ZnFe_2_O_4_ (JCPDS card 00–002-1043). Bragg reflections associated to ZnO secondary phase can also be detected in the initial ZnFe_2_O_4_ sample. The zinc ferrite crystallite size calculated according to the Scherrer equation was about 9 nm (see Table 1). The XRD pattern of the 2D/2D TiO_2_-GO composite revealed similar diffraction peaks of anatase nanoparticles, with the most intense at 2θ = 25.25° (JCPDS card no. 21–1272). The amount of graphene oxide relative to TiO_2_ was 1%. Therefore, the typical diffraction signals for graphene oxide were undetected in the XRD pattern of TiO_2_-GO and TiO_2_-GO-ZnFe_2_O_4_. In the previous studies, the characteristic diffraction peaks of GO at 2θ 10–12° for TiO_2_/GO composites were also not observed since its amount was negligible compared to TiO_2_, and its diffraction intensity was relatively low (Saravani et al. 2019; Yadav and Kim 2016; Štengl et al. 2013). The crystallite size of anatase in TiO_2_-GO and TiO_2_-GO-ZnFe_2_O_4_ was about 22–23 nm, indicating that the incorporation of GO in the TiO_2_ structure has little influence on the crystallite size of the TiO_2_ phase structure.

For 2D/2D TiO_2_-GO-ZnFe_2_O_4_ composite, all the characteristic diffraction peaks can be assigned to ZnFe_2_O_4_ or anatase TiO_2_, confirming the combination of ZnFe_2_O_4_ with GO-TiO_2_. The most intense diffraction peak of zinc ferrite particles in the XRD pattern of 2D/2D TiO_2_-GO-ZnFe_2_O_4_ composite became weaker than that of ZnFe_2_O_4_ due to even distribution and relatively lower content in the final composite structure. The BET surface area varied from 11.5 m^2^·g^−1^ to 24.5 m^2^·g^−1^.

**Fig. 2:**
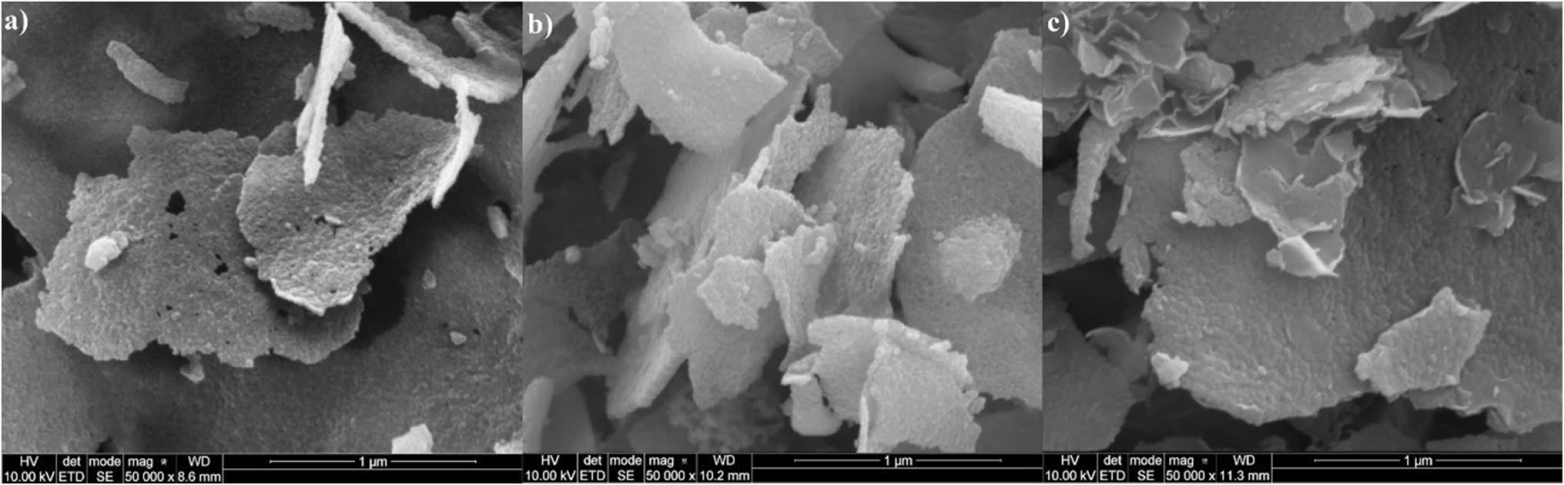
SEM analysis of TiO_2_ nanosheets after lyophilization (**a** and **b**) and after calcination at 700 °C (**c**)

The morphology of the obtained 2D TiO_2_ was examined by SEM microscopy analysis. Based on that, it can be concluded that TiO_2_ obtained by lyophilization is characterized by a two-dimensional structure, as presented in Fig. 2.

**Fig. 3:**
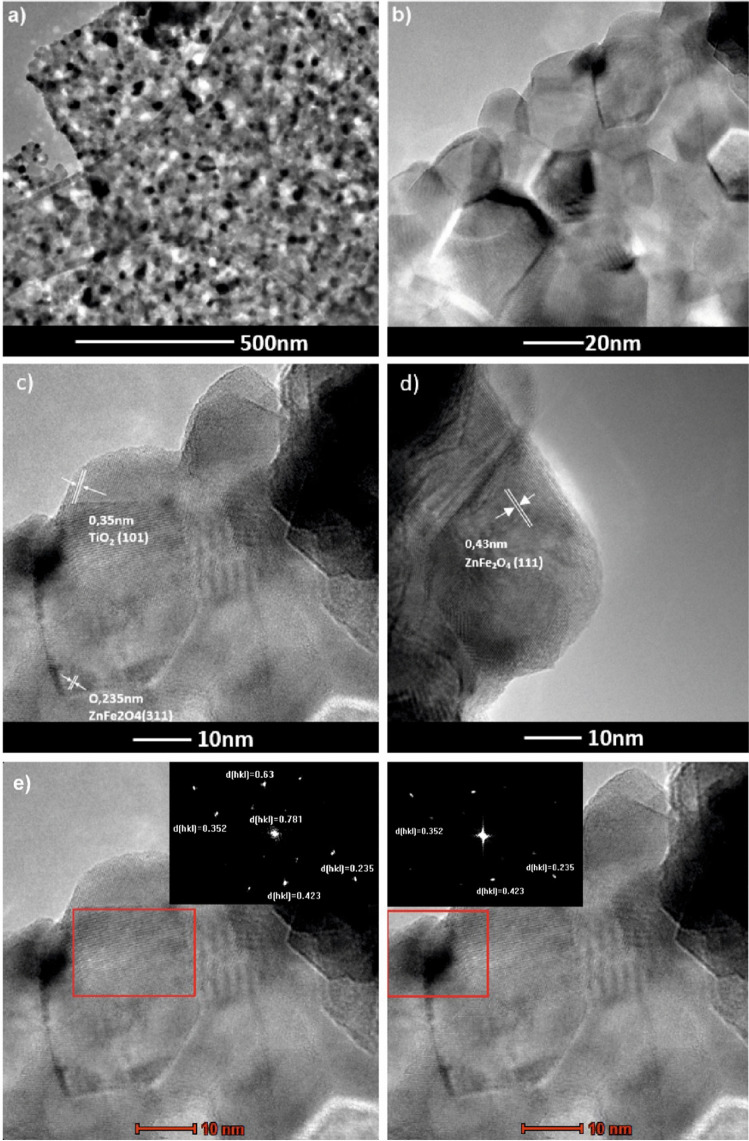
2D/2D TiO_2_-GO-ZnFe_2_O_4_ composite (**a** and **b**) TEM images, (**c** and **d**) HRTEM with marked lattice spacing for TiO_2_ and ZnFe_2_O_4_, and (**e**) TEM images of composite with fast Fourier transform results (inset)

**Fig. 4:**
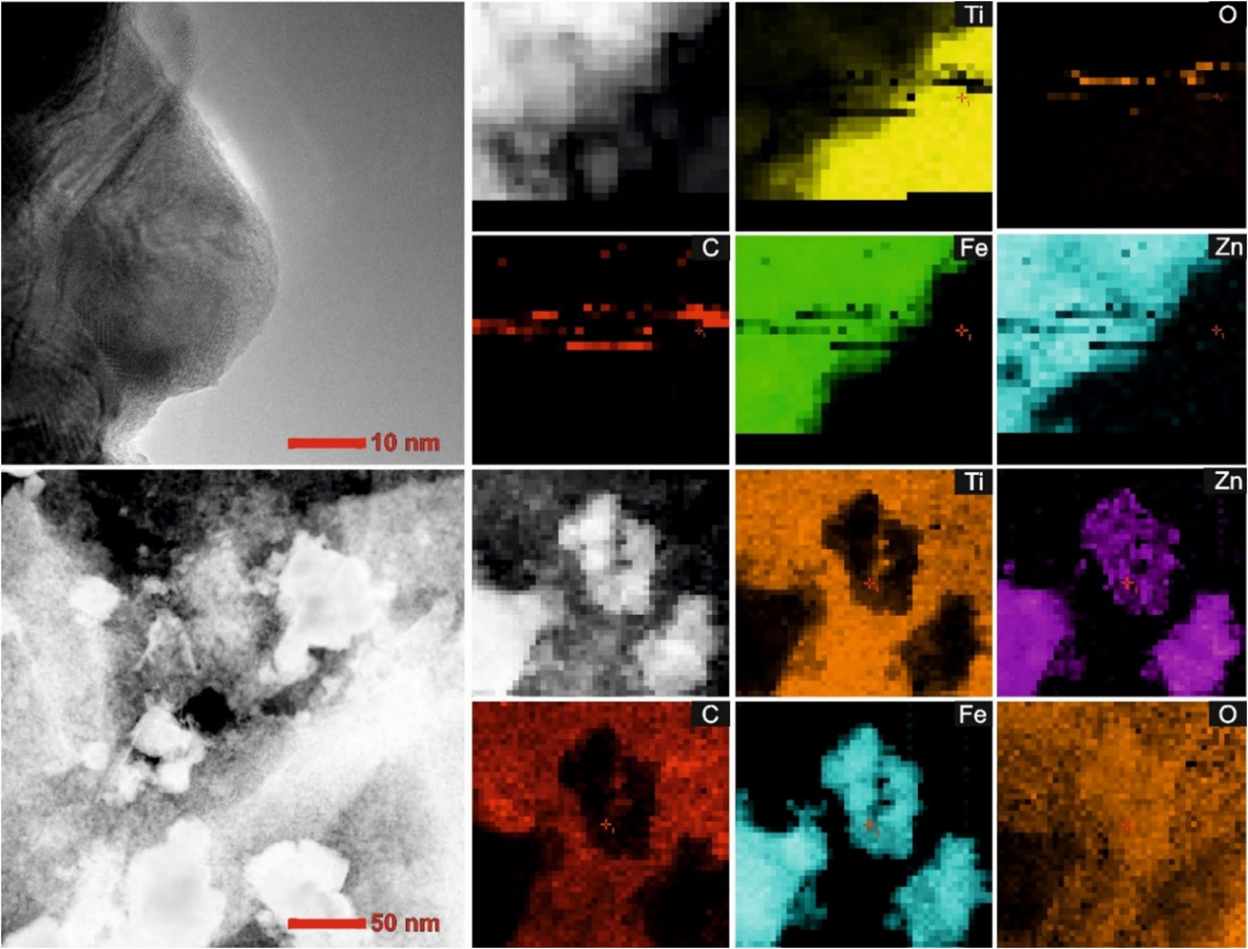
Morphology (TEM images) and mapping of the 2D/2D TiO_2_-GO-ZnFe_2_O_4_ photocatalyst

The TEM microscopic analyses confirmed the formation of a 2D/2D TiO_2_-GO-ZnFe_2_O_4_ nanocomposite structure with a total particle size of the composite of about 30–40 nm (Fig. 3a and b). The particles are flaky (2D structure) and contain GO embedded with zinc ferrite. The layer spacing equal to 0.35 nm (Fig. 3c) referred to (101) planes of TiO_2_. For 2D/2D TiO_2_-GO-ZnFe_2_O_4_ composite, the lattice spacings of 0.235 nm and 0.423 nm were also distinguished, indicating the presence of (311) and (111) planes of ZnFe_2_O_4_. Moreover, lattice fringes from the HRTEM results also give additional information about inter-planar distance d_002_ for GO equaled 0.71 nm (Mukhopadhyay et al. 2016; Lemine et al. 2011). Based on STEM images and mapping of the selected area, graphene oxide layers are intercalated in 2D TiO_2_ and embedded with ZnFe_2_O_4_ (see Fig. 4). Energy dispersion spectroscopy (EDS) confirmed the presence of Zn, Fe, C, O, and Ti in the structure of the photocatalytic material. Signals for Zn and Fe occurred in the same area inside the structure, and signals for C, O, and Ti outside the magnetic particle structure. In the composite, graphene oxide consists of several layers.

**Fig. 5:**
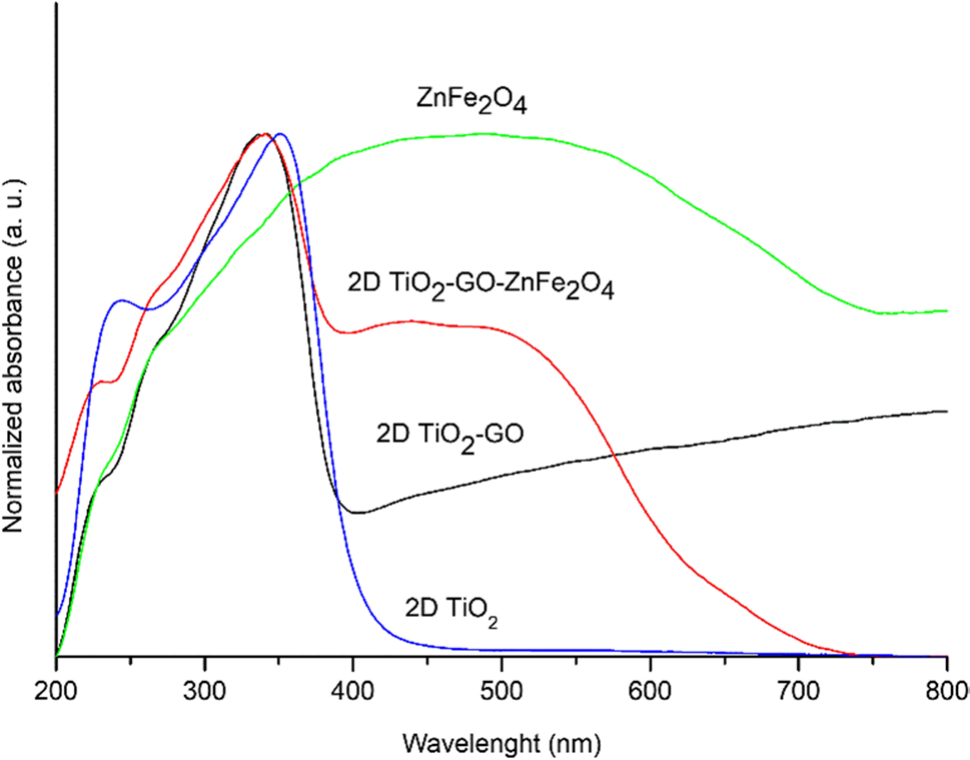
Diffuse reflectance spectra of ZnFe_2_O_4_, pure 2D TiO_2_, 2D TiO_2_-GO, and 2D/2D TiO_2_-GO-ZnFe_2_O_4_ composite

The optical absorption properties of the magnetic photocatalysts are presented in Fig. 5. The analyzed samples absorb UV light due to the sp-d interaction between valence band electrons of O and d electrons of Ti or Zn/Fe atoms in TiO_2_ and ZnFe_2_O_4_ structures, respectively. The light absorption edge in the ultraviolet region for 2D titania particles was about 400 nm. For 2D TiO_2_-GO and TiO_2_-GO-ZnFe_2_O_4_ composites, the absorption was markedly redshifted after incorporating ZnFe_2_O_4_ with GO and TiO_2_ layer. The ternary composite absorbs light in UV–vis light; therefore, solar light can be used for photocatalytic degradation of emerging contaminants in the presence of 2D/2D TiO_2_-GO-ZnFe_2_O_4_ particles. Obtained spectra were transformed into the Kubelka–Munk function, and the Tauc transformation was used to determine the optical bandgap energy. As expected, the ZnFe_2_O_4_ ferrite sample exhibited low bandgap energy of 1.5 eV, which widened after hydrothermal treatment with GO. The bandgap energy for 2D TiO_2_ was 3.2 eV.

According to the literature, the energy bandgap of pure TiO_2_ particles depends on the polymorphs and is reported at ~ 3.0 eV for rutile and 3.2–3.3 eV for anatase. The energy bandgap for 2D TiO_2_-GO was 2.9 eV. The extended light absorption in the visible light of TiO_2_-GO can be ascribed to the formation of Ti–O-C covalent bonds between oxygen groups from GO and Ti atoms promoted during the hydrothermal treatment (Ti–O–C). The energy bandgap of TiO_2_-GO composite with magnetic particles of ZnFe_2_O_4_ was about 2.85 eV.

**Fig. 6:**
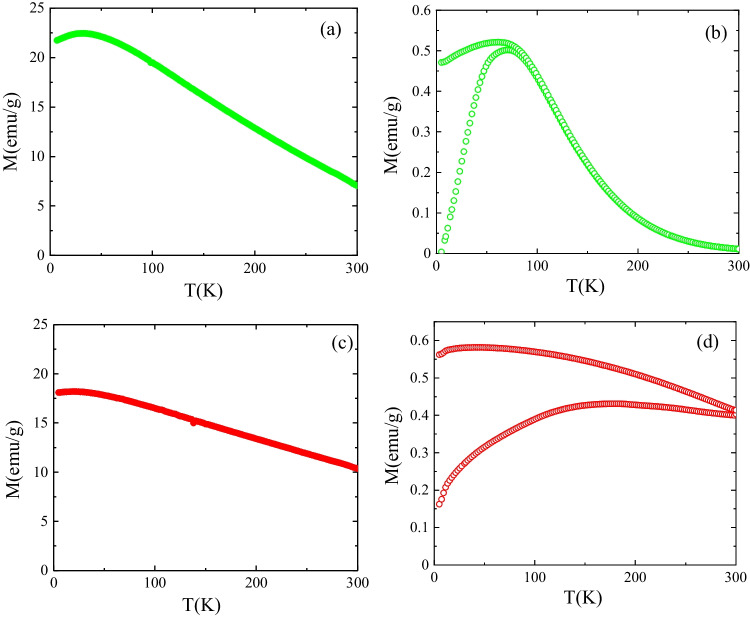
High field magnetization, *M*, (applied magnetic field *µ*_*0*_*H* = 6 T) versus temperature, *T*, for (**a**) ZnFe_2_O_4_ nanoparticles and (**c**) 2D TiO_2_-GO-ZnFe_2_O_4_ composite. Zero-field-cooled and field-cooled (*ZFC–FC*) magnetization curves obtained at 50 Oe for (**a**) ZnFe_2_O_4_ nanoparticles and (**d**) 2D TiO_2_-GO-ZnFe_2_O_4_ composite

Figure 6 shows the high field magnetization, *M*, (applied magnetic field *µ*_*0*_*H* = 6 T) versus temperature, *T*, and zero-field-cooled and field-cooled (ZFC–FC) magnetization curves obtained at 50 Oe for the ZnFe_2_O_4_ nanoparticles and the 2D TiO_2_-GO-ZnFe_2_O_4_ composite. Bulk ZnFe_2_O_4_ is antiferromagnetic with Nèel temperature, *T*_*N*_ = 10 K, displaying reduced magnetic susceptibility at 300 K. Bulk Zn ferrite is a normal spinel, where Fe^3+^ cations anti-ferromagnetically coupled occupy octahedral (*B*) sites, and Zn^2+^ cations (non-magnetic) are preferentially located at the tetrahedral *A* positions (Antic et al. 2013). However, ferrimagnetic behavior is usually found at the nanoscale as a consequence of the cation redistribution between octahedral and tetrahedral sites and the occurrence of mixed states (i.e., Fe^3+^ cations in both *B* and A sites). This behavior can be confirmed in Fig. 6a, where the high field magnetization evolves with temperature similarly to reported ZnFe_2_O_4_ ferrimagnetic nanoparticles (Gómez-Polo et al. 2018).

Furthermore, the occurrence of a maximum value in the *ZFC* magnetization curve around 75 K (see Fig. 6a) confirmed the superparamagnetic behavior of the Zn ferrite nanoparticles with characteristic blocking temperature, *T*_*B*_ ∙ 75 K. However, slight changes in the magnetic response are found in the 2D TiO_2_-GO-ZnFe_2_O_4_ composite, mainly in the *ZFC–FC* magnetisation curves (see Fig. 6d).

**Fig. 7:**
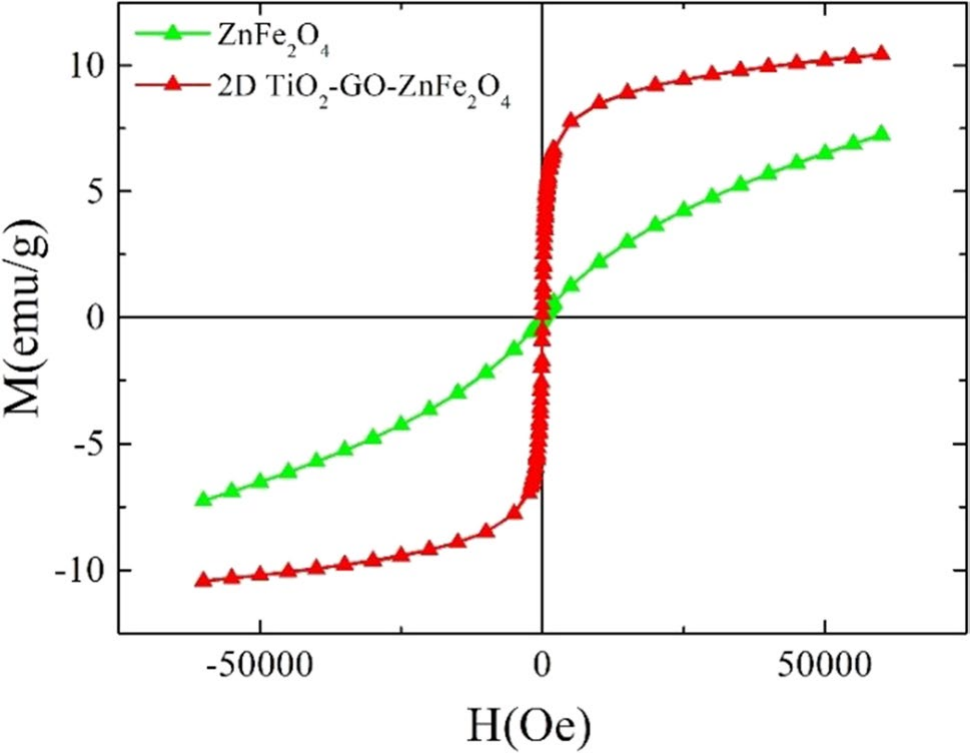
Room temperature *M-H* hysteresis loops ZnFe_2_O_4_ nanoparticles and (**c**) 2D TiO_2_-GO-ZnFe_2_O_4_ composite

The shift of the wide maximum in the *ZFC* magnetization toward higher temperatures would be compatible with wide size distribution and an increase in the mean nanoparticle size. Nevertheless, no significant changes can be detected in the high-field magnetization (see Fig. 6c) compared with the initial ZnFe_2_O_4_ nanoparticles. It should be noted that the superparamagnetic behavior (anhysteric magnetic behavior at temperatures above *T*_*B*_) is of relevance considering the photocatalytic applications since it enables a better dispersion of the nanoparticles due to the absence of inter-particle magnetic interactions. Additionally, a high magnetic susceptibility facilitates nanoparticle extraction from the medium in which they are immersed through the application of an external magnetic field. In the present samples, anhysteretic *M-H* at 300 K hysteresis loops can be deduced from Fig. 7 in the initial ZnFe_2_O_4_. However, for the 2D TiO_2_-GO-ZnFe_2_O_4_ composite, non-zero coercivity (*H*_*c*_ ≈ 30 Oe) and magnetization at the remanence (*M*_*r*_ ∙ 0.4 emu/g), close to the SQUID detection limit, are detected and ascribed to the larger magnetic ferrite nanoparticles. However, these low values, close to the anhysteretic response, confirm the utility of the composite for photocatalytic applications (i.e., reduced magnetic inter-particle interactions).

Furthermore, the remarkable increase in the magnetic susceptibility (i.e., magnetization at low applied magnetic fields) in this composite sample would enhance the recycling and magnetic separation capability of the photocatalyst.

### Photocatalytic degradation of ibuprofen in the presence of 2D/2D TiO_2_-GO-ZnFe_2_O_4_ nanocomposites

**Fig. 8:**
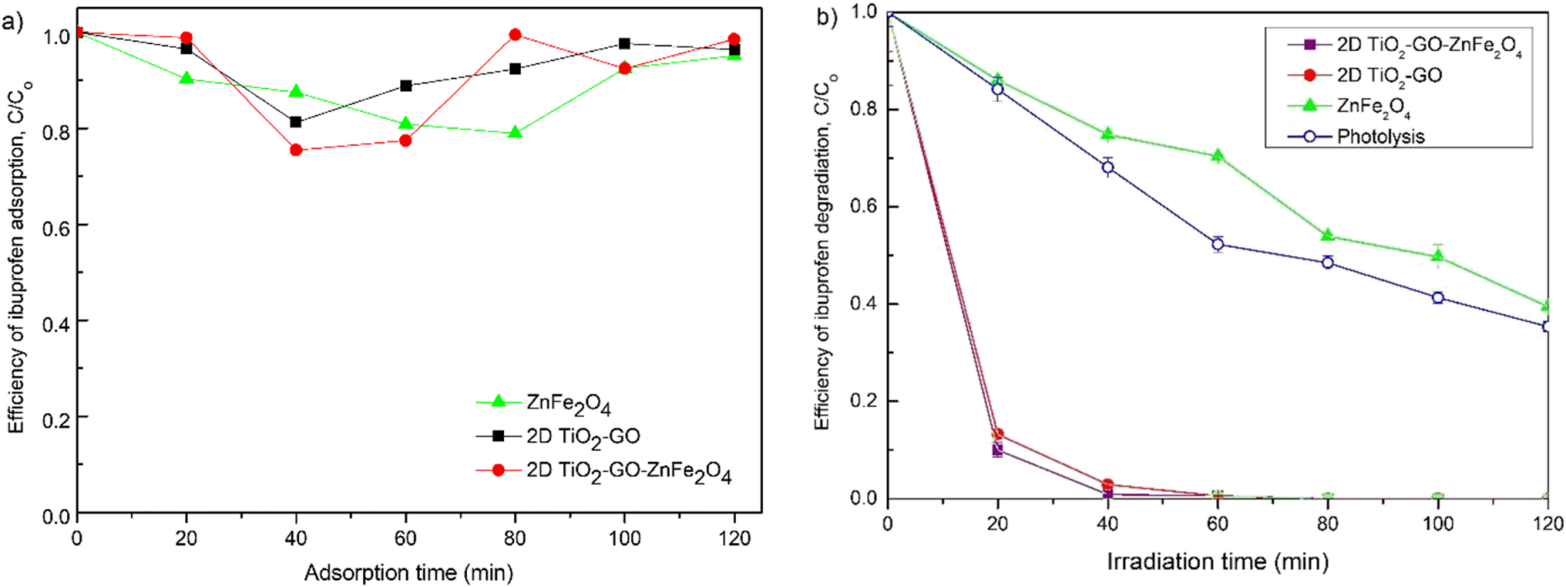
The efficiency of ibuprofen degradation in the photocatalytic reaction under UV–vis light irradiation

Chemical adsorption takes place in graphene-based materials. This adsorption mechanism is based on the interaction with π-electron of the graphitic rings, π-π interaction, and hydrogen bond (Liu et al. 2020; Guedidi et al. 2013; Low et al. 2017). Therefore, before the photocatalytic analysis, the adsorption properties of the composite materials were studied and presented in Fig. 8a. The obtained photocatalysts revealed relatively low adsorption of ibuprofen during 120 min of the process. Besides, no relationship was noticed between the ability of photocatalysts to adsorb organic pollutants and the specific surface area.

The photodegradation properties of ZnFe_2_O_4_ were similar to ibuprofen depletion during photolysis. The highest photocatalytic degradation was observed for TiO_2_-GO nanosheets and 2D TiO_2_-GO-ZnFe_2_O_4_ composite. The photocatalytic decomposition of ibuprofen reached 90% in only 20 min of photodegradation under simulated solar light (see Fig. 8b). During the next 20 min (after 40 min of irradiation), about 99% and 97% were degraded for 2D TiO_2_-GO-ZnFe_2_O_4_ and 2D TiO_2_-GO, respectively. The combination of 2D TiO_2_-GO with zinc spinel ferrite does not deteriorate the photocatalytic properties and allows to take advantage of high photocatalytic activity under solar light and magnetic separation properties. In this regard, the hybrid nanocomposite of 2D/2D TiO_2_-GO-ZnFe_2_O_4_ determines photocatalyst new and improved properties.

**Fig. 9:**
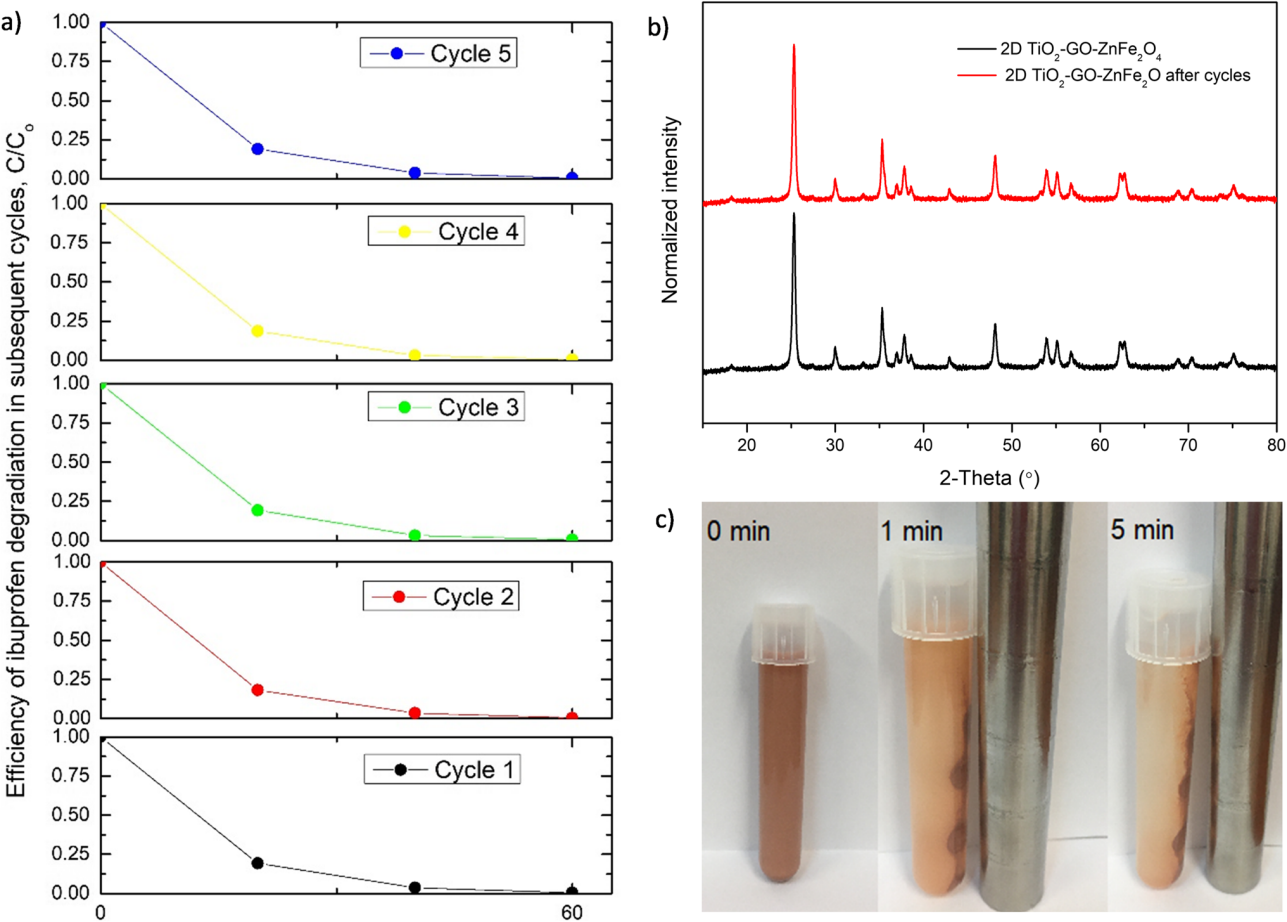
The efficiency of ibuprofen degradation in the presence of 2D TiO_2_-GO-ZnFe_2_O_4_ photocatalyst measured in the five subsequent cycles of degradation (**a**), XRD diffractograms of 2D/2D TiO_2_-GO-ZnFe_2_O_4_ photocatalyst before and after the subsequent cycles of degradation (**b**), and magnetic photocatalyst separation after 1 min and 5 min (**c**)

Furthermore, the 2D/2D TiO_2_-GO-ZnFe_2_O_4_ composite material was selected for the reusability studies. The five subsequent cycles were performed to examine the photocatalytic stability of the magnetic photocatalyst after its recovery, as presented in Fig. 9a.

After each run of ibuprofen degradation, a defined amount of IBU was added to the reactor to attain the initial concentration of 21 mg/dm^3^; then, next photodegradation cycle was started. This procedure mitigates the risk of the impact of photocatalyst amount change or kinetics change due to fresh/new photocatalyst add-ons or any potential photocatalyst surface changes during the drying process. Therefore, it can be understood as “perfect separation and reuse” of photocatalyst. The 2D/2D TiO_2_-GO-ZnFe_2_O_4_ composite showed excellent stability without any changes in the structure of the composite material in the subsequent cycles of photocatalytic degradation (Fig. 9a and b). High photocatalytic degradation efficiency was maintained after the fifth subsequent cycle without any significant changes in ibuprofen degradation and mineralization. The TOC reduction TOC_o_-TOC_t_/TOC_o_ for each cycle amounted to 0.62 ± 0.04. After the fifth irradiation cycle, the photocatalyst was magnetically separated (Fig. 9c). The use of zinc ferrite as a magnetic material allowed the separation of more than 96% of the entire photocatalyst nanoparticles from the suspension in less than 5 min, including loss of photocatalyst during drying before use in the next cycle.

**Fig. 10:**
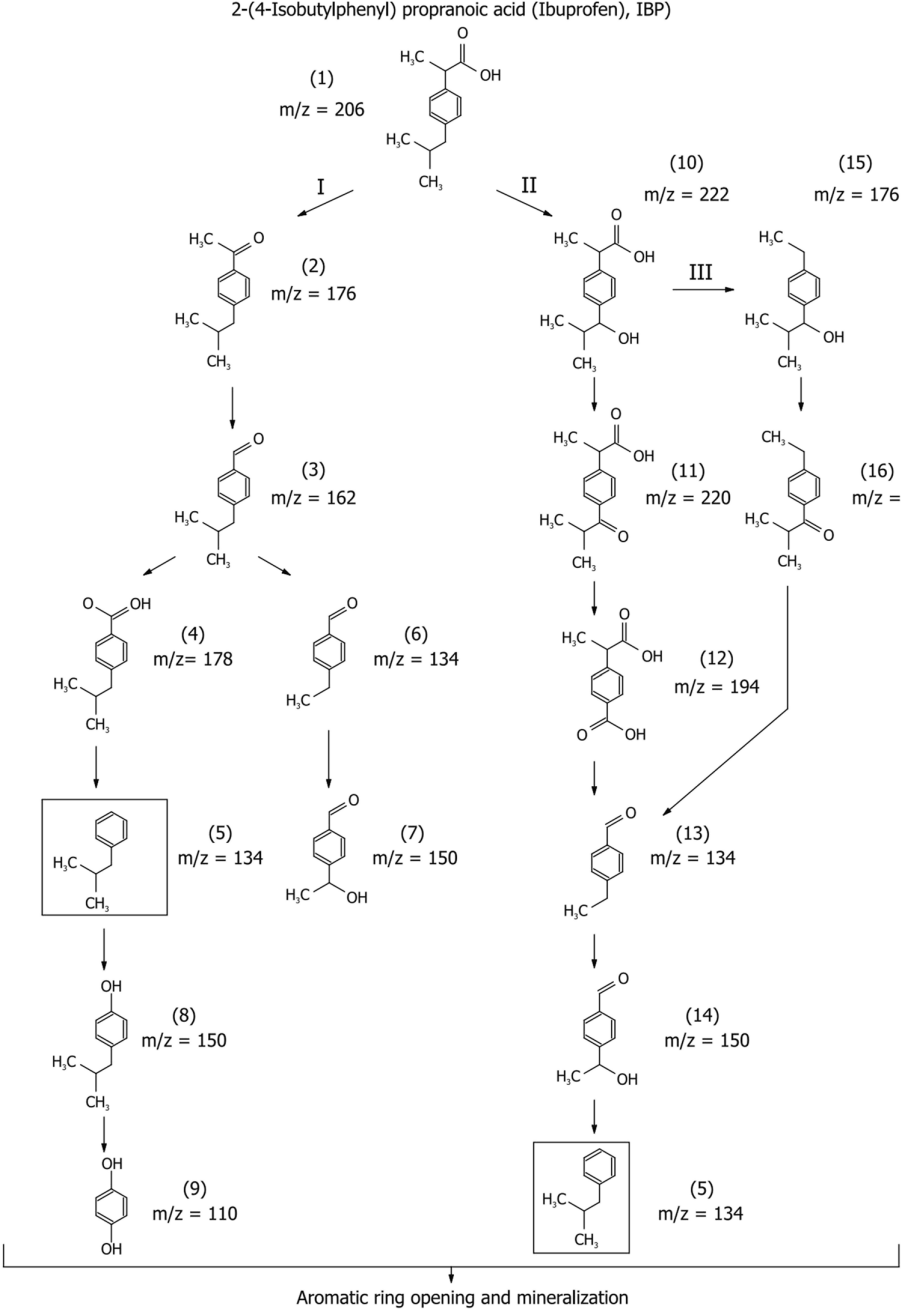
The possible ibuprofen degradation pathways in the presence of 2D/2D TiO_2_-GO-ZnFe_2_O_4_ photocatalyst under simulated solar light

Further LC–MS analyses were performed to identify reaction intermediates and understand the mechanism of IBU degradation during the photocatalytic process. The results showed that 2D TiO_2_-GO and 2D TiO_2_-GO-ZnFe_2_O_4_ photocatalysts effectively degraded IBU. The phenolic compounds or aromatic carboxylic acids were detected as intermediates of ibuprofen photodegradation. Molecular structures for the intermediate were proposed based on ion molecular weights and MS fragmentation patterns. The proposed pathways of IBU degradation are presented in Fig. 10. First, 2-(4-isobutylphenyl) propanoic acid (ibuprofen, IBU) hydroxylation results in the formation of 2–4-isobutylacetophenone (**2**). Further demethylation leads to the loss of the terminal –CH_3_ group producing 4-isobutylbenzaldehyde (**3**), which can be hydroxylated to carboxylic acid derivative (**4**), or its isobutyl group can be demethylated to 4-ethylbenzaldehyde (**6**) and 4-(1-hydroxyethyl)benzaldehyde (**7**). Finally, through the decarboxylation yielding to isobutylbenzene (**5**), which is hydroxylated to the phenolic derivative (**8**) and (**9**). Then, oxidation leads to aromatic rings opening by the radical attack and producing short-chain organic acids, further mineralizing to CO_2_ and H_2_O. In pathway II, 2-(4-isobutylphenyl) propanoic acid (**1**) is hydroxylated to 2-(4-(1-hydroxy-2-methyl propyl)phenyl)propanoic acid (**3**) and further transformed into the carboxylic acid derivative (**12**). The decarboxylation and isobutyl group demethylation give (**13**) further hydroxylated to form (**14**). Finally, the decarboxylation leads to the formation of isobutylbenzene, which is hydroxylated to the phenolic derivatives. The third pathway of IBU degradation is direct demethylation. In the first stage, the IBU is hydroxylated, and 2-(4-(1-hydroxy-2-methyl propyl)phenyl)propanoic acid is formed. Subsequently, decarboxylation of this intermediate leads to the formation of 1-(4-ethyl phenyl)-2-methyl propan-1-ol (**15**), which is further demethylated and hydroxylated to form isobutylbenzene (**5**). Finally, the reactive oxygen species are involved in degradation, which attacks the phenyl ring yielding simple aromatic organic compounds generation, e.g., hydroquinone. Then, the phenyl ring in these compounds disintegrates, and short-chain organic acids are produced, which are further mineralized to CO_2_ and H_2_O.

### Identification of reactive species and mechanism of ibuprofen photodegradation

**Fig. 11:**
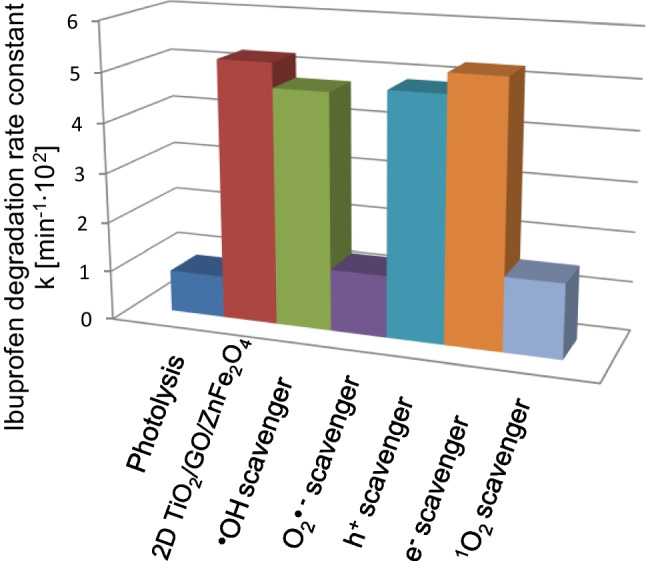
Photocatalytic degradation of IBU for 2D TiO_2_-GO-ZnFe_2_O_4_ photocatalyst in the presence of **·**OH**, ·**O_2_^−^, h^+^, e^−^, and ^1^O_2_ scavengers

In order to confirm the mechanism of ibuprofen photocatalytic degradation, reactive oxygen species participating in the photocatalytic process were analyzed in reference experiments in the presence of scavengers. In this regard, the photocatalytic activity of the most active 2D/2D TiO_2_-GO-ZnFe_2_O_4_ photocatalyst in the presence of tert-butanol (hydroxyl radical scavenger, **·**OH), benzoquinone (superoxide radical scavenger, **·**O_2_^−^), sodium azide (singlet oxygen, ^1^O_2_^−^ scavenger), ammonium oxalate (h^+^ scavenger), and silver nitrate (e^−^ scavenger) was investigated. Photocatalytic degradation of ibuprofen determined without scavengers served as the reference sample. The introduction of t-BuOH, which captures photogenerated hydroxyl radicals, slightly affected the efficiency of IBU degradation (see Fig. 11). However, the photocatalytic efficiency was significantly reduced in the presence of benzoquinone and sodium azide, indicating that **·**O_2_^−^ and ^1^O_2_ play an important role in the photodegradation of ibuprofen.

**Fig. 12:**
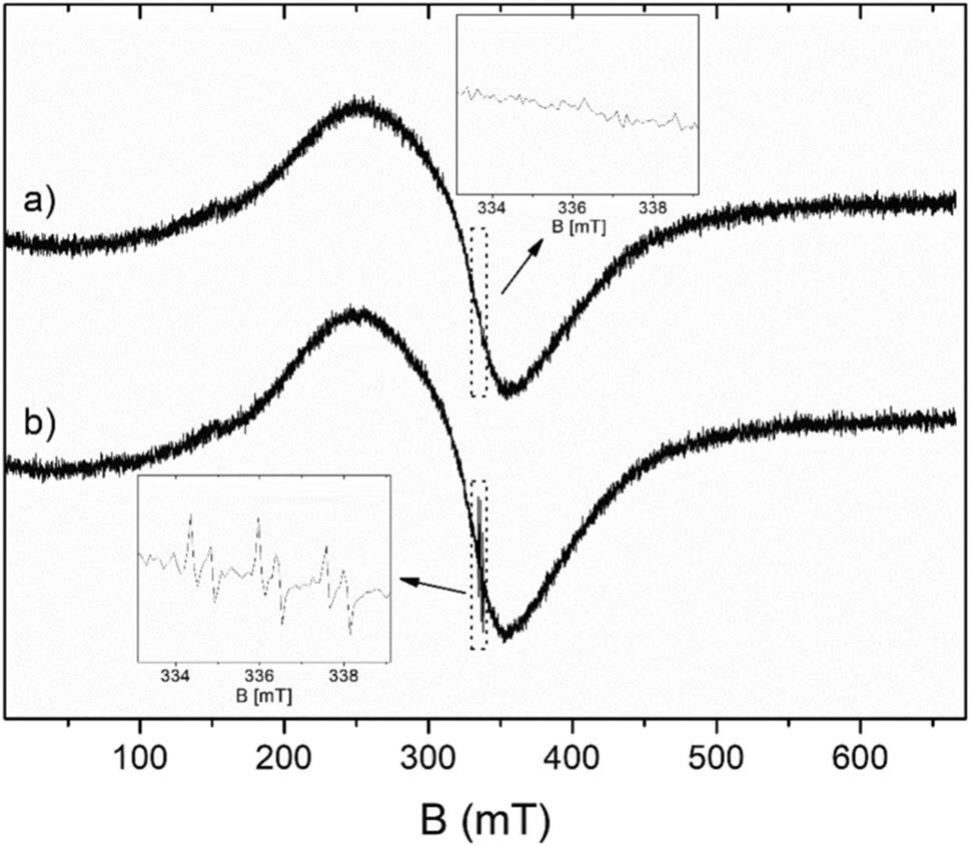
ESR spectra of the aqueous suspension of 2DTiO_2_/GO/ZnFe_2_O_4_ performed under aerobic conditions in the presence of PBN spin trap. (**a**) Non-irradiated sample and (**b**) irradiated sample. The inserts show the parts of the spectra near the g-factor 2.0056

Electron spin resonance is one of the most sensitive methods for free radicals detection. However, superoxide or hydroxyl radicals have too short lifetime to detect such radicals directly by ESR. In order to obtain a more stable adduct that could be detected and identified by ESR, PBN as a spin trap for short-lived radicals was used. The broad range of ESR spectra of the sample before irradiation and several dozen minutes after the end of irradiation are presented in Fig. 12. For irradiated and non-irradiated samples, the spectra consist of broad, non-symmetrical line characteristic of ferrite nanoparticles (Li et al. 1996; Naseri et al. 2011; Coskun and Korkmaz 2014; Naseri et al. 2014). As presented in the inserts of Fig. 12, the lines which can be assigned to radicals appear only in the spectrum of the irradiated sample.

**Fig. 13:**
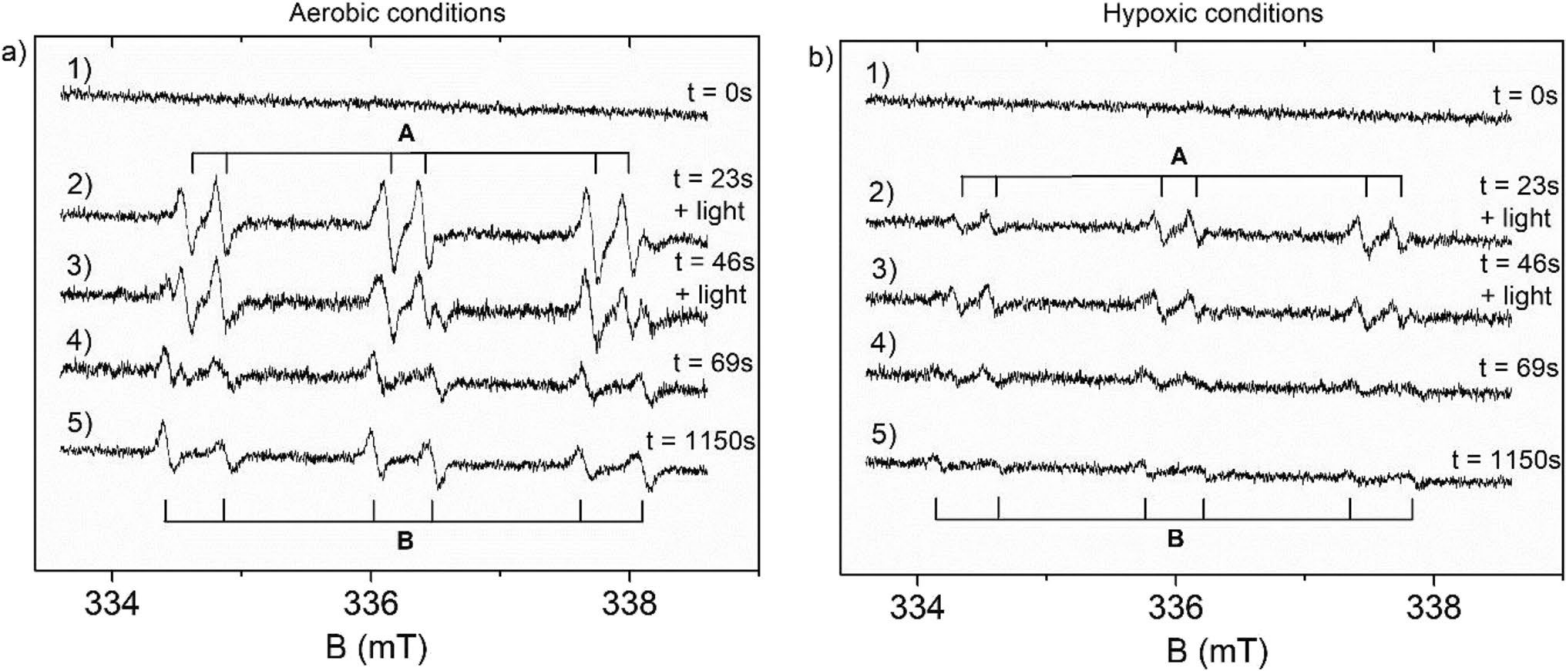
ESR spectra recorded under aerobic (**a**) and hypoxic conditions for (1) non-irradiated sample, (2–3) spectra recorded during light irradiation, (4–5) spectra recorded after irradiation was finished. The recording time of each spectrum was approximately 23 s. The numbers on the right side of the figure represent the starting time of each record

Furthermore, the ESR spectra were analyzed under aerobic conditions (Fig. 13a) and hypoxic conditions (Fig. 13b). Without light exposure, the presence of any radicals in the sample was not observed (spectrum 1 in Fig. 13a and b). Within tens of seconds of irradiation, a gradual increase of ESR lines was observed (spectra 2 and 3 in Fig. 13a and b), and a distinct triplet of doublets was formed (radical A), which indicated the interaction of an unpaired electron with a nitrogen nucleus and a proton. The radical A was described by the spin Hamiltonian parameters presented in Table 2 and can be attributed to PBN-OH radical adduct (Dodd and Jha 2011; Tero-Kubota et al. 1982; Harbour et al.^1974^). This radical adduct is created if a short-lived hydroxyl radical is trapped by PBN. Even during irradiation, the second paramagnetic complex B appears in the spectrum (see the last small line in spectra 3, Fig. 13a and b). Upon termination of irradiation (spectra 4 and 5 in Fig. 13a and b), the spectrum continues to change, and the intensity of paramagnetic complex B increases and complex A decreases. This is evidence of the conversion of complex A into complex B. About one thousand seconds after the end of irradiation, only the B radical was released into the spectrum. This is believed that the stable complex B is the benzoyl spin adduct, resulting from the decomposition of PBN (Dodd and Jha 2011; Tero-Kubota et al. 1982).

**Table 2:** Hyperfine constants of nitrogen (A^N^) and hydrogen (A^H^), and g-factor for radicals created after UV irradiation

Radical adduct	*A*^*N*^ (mT)	*A*^*H*^ (mT)	g-factor
A	1.56 ± 0.01	0.27 ± 0.01	2.0057 ± 0.0001
B	1.60 ± 0.01	0.46 ± 0.01	2.0056 ± 0.0001

It should be noted that for both cases, i.e., hypoxic and aerobic conditions, ESR detected the same kinds of paramagnetic centers: hydroxyl radical and benzoyl spin adduct. However, the ESR spectra of the sample recorded under aerobic conditions (Fig. 13a) are at least twice as intense as those corresponding to hypoxic conditions (Fig. 13b).

Photoexcitation of TiO_2_ can create a valence band hole *h*_*vb*_^+^ and *e*_*eb*_^*−*^ pair (A):

1 $$TiO_{2\;}{\underrightarrow{hv\;}\;TiO}_2\left(h_{vb}^++e_{eb}^-\right)$$

In the presence of water molecules or hydroxide ions, the positive holes can generate hydroxyl radicals, while the conduction band electrons change the oxidation state of titanium ions (Dodd and Jha 2011; Cho et al. 2005):

2 $$h_{vb^+}+H_2O\rightarrow H^++\cdot OH$$

3 $$h_{vb^{+}}+{OH}^{-}\to \cdot OH$$

4 $$e_{eb^{-}}+{Ti}^{4+}\to {Ti}^{3+}$$

Under aerobic conditions, superoxide radicals are created (Eq. 5), which can be transformed into hydrogen peroxide (Eq. 6) or hydroxyl radicals (Eq. 7). Moreover, Eq. 8 indicates that the hydroxyl radical may be formed by the interaction of hydrogen peroxide with a conduction band electron.

5 $${O}_{2}+e_{eb^{-}}\to {O}_{2}{\cdot }^{-}$$

6 $${O}_{2}{\cdot }^{-}+e_{eb^{-}}+{2H}^{+}\to {H}_{2}{O}_{2}$$

7 $${O}_{2}{\cdot }^{-}{H}_{2}{O}_{2}\to \cdot OH+{OH}^{-}+{O}_{2}$$

8 $${e}_{eb}^{-}+{H}_{2}{O}_{2}\to \cdot OH+{OH}^{-}$$

9 $${O}_{2}{\cdot }^{-}+{h}_{vb^{+}}\to {}^{1}{O}_{2}$$

The above difference in the ESR spectra intensity strongly suggests that superoxide radicals are generated by irradiation, but their lifetime is too short to be trapped by PBN and detected by ESR. The higher intensity of the ESR spectrum for the sample under aerobic conditions is due to the transformation of superoxide radicals to hydroxyl radicals, as described directly by Eq. 7 and indirectly by Eq. 6 and 8.

**Fig. 14:**
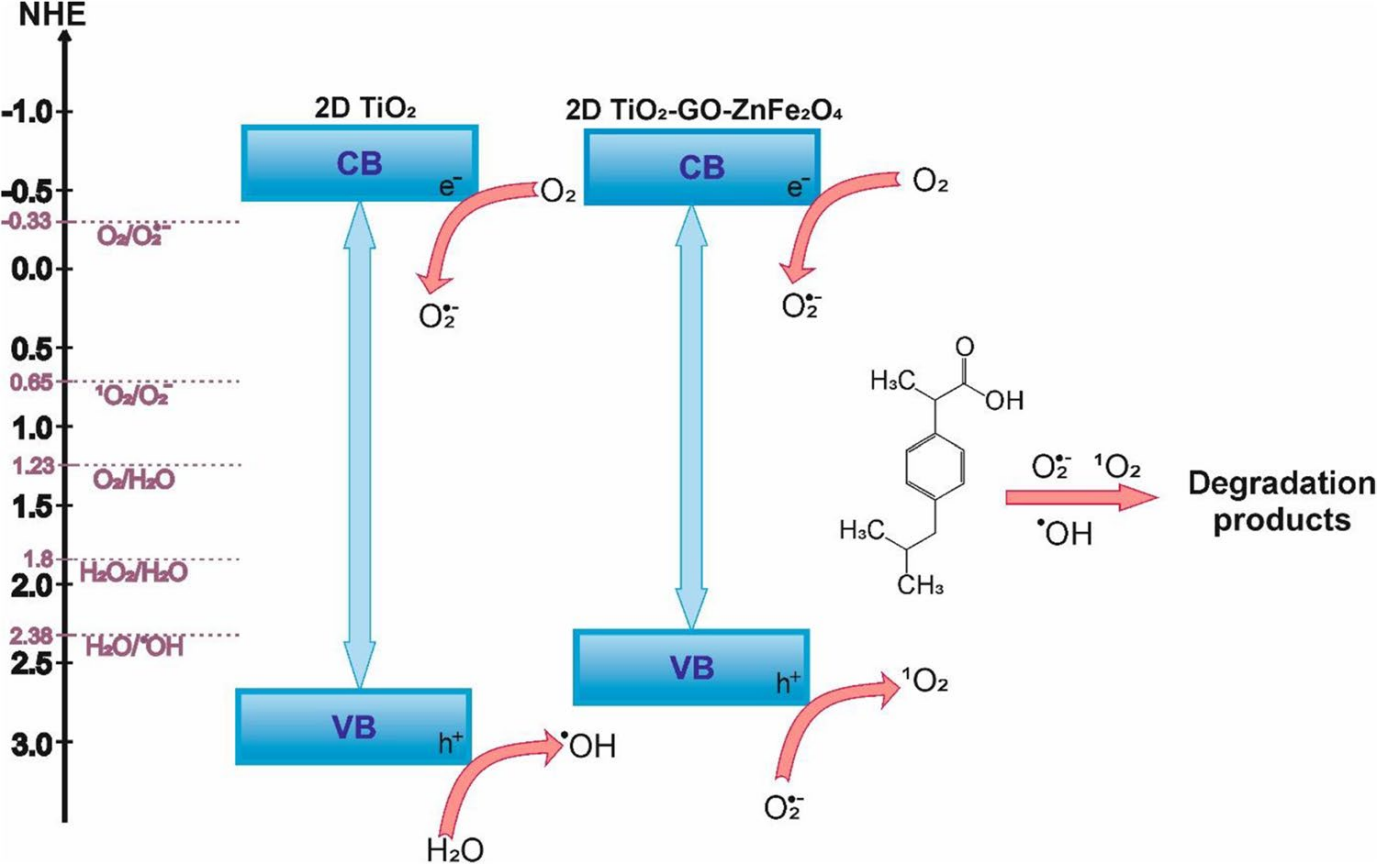
Schematic representation of 2D TiO_2_ and 2D/2D TiO_2_-GO-ZnFe_2_O_4_ band structure and excitation under UV–vis light

Figure 14 shows a graphical presentation of the photocatalyst band edges position and charge transfer during excitation with a light greater than its bandgap energy. For n-type semiconductors, the flat band potential is almost equal to the conduction band potential. The flat band potential of 2D TiO_2_-GO-ZnFe_2_O_4_ was -0.56 V vs. NHE. The valence band edge location estimated according to a value of the flat band edge position and bandgap energy was 2.34 V vs. NHE.

According to the obtained results, 2D/2D TiO_2_-GO-ZnFe_2_O_4_ can effectively oxidize H_2_O to oxygen and reduce oxygen into superoxide radicals (Eq. 5). Furthermore, singlet oxygen can be produced by the oxidation of the superoxide radical anion (Eq. 10). The band structure of the 2D TiO_2_-GO-ZnFe_2_O_4_ composite correlates with significant inhibition of ibuprofen degradation in the presence of benzoquinone as a **˙**O_2_^−^ scavenger and sodium azide as a ^1^O_2_ scavenger, suggesting that the degradation mechanism involving superoxide radicals and singlet oxygen plays a crucial role. Furthermore, the presented results correspond with the electronic spin resonance ESR analysis, indicating the significant involvement of superoxide radicals in the IBU photodegradation. The graphene oxide CB band position is below the 2D TiO_2_ CB position (Low et al. 2017; Xiang et al. 2011). Therefore, the intercalation of graphene oxide in the structure of 2D TiO_2_-based composite enhances the photocatalytic activity of TiO_2_ due to the fast migration of conduction band electrons from the photocatalyst to graphene oxide. The transfer of e^−^ elongates the lifetime of holes in the VB of TiO_2_. Accumulated electrons participate in superoxide anion radical generation from oxygen, whereas photogenerated holes participate in singlet oxygen production as a main reactive oxygen species taking part in the photocatalytic oxidation reaction.

### Toxicity assessment

According to the Globally Harmonized System of Classification and Labeling of Chemicals, ibuprofen is classified as a compound that is harmful to aquatic organisms (Ortiz de García et al. 2014). The toxicity of IBU degradation products may differ from the initial compound. Therefore, it is necessary to examine the toxicity of the IBU solution during the photocatalytic process. The solutions of IBU photodegradation were tested for their toxicity toward *Vibrio fischeri*. The EC_50_ obtained for the initial IBU solution was equal to 59.05%, which leads to EC_50_ = 11.81 mg·dm^3^. The determined EC_50_ value of IBU toxicity toward *Vibrio fischeri* bacteria complies with literature data EC_50_ = 12.1 mg·dm^3^ (Ortiz de García et al. 2014). The following TU_50_ value was equal to 8.467, qualifying the IBU solution as a toxic compound following the toxic unit classification (Dökmeci et al. 2014).

The photolytic treatment caused an increase in the toxicity of IBU solution. After 120 min of IBU photolysis under UV–vis irradiation, the EC_50_ value of the remaining solution reached 32.47%. Based on the IBU degradation efficiency for photolysis (IBU degradation efficiency equaled 64%), the removal of pharmaceutical during the photolytic process is incomplete. IBU residue, combined with toxic by-products, increased the reaction mixture toxicity.

The photocatalytic process in the presence of 2D TiO_2_-GO-ZnFe_2_O_4_ resulted in a significant decrease in the toxicity of the solution, reaching an EC_50_ value equal to 98.29%. The residual toxicity of the examined sample can be caused by trace amounts of by-products such as 1-(4-ethyl phenyl)-2-methyl propan-1-ol, whose presence was confirmed by LC–MS analysis.

## Conclusions

In summary, the 2D/2D TiO_2_-GO-ZnFe_2_O_4_ magnetic photocatalyst was successfully synthesized using the fluorine-free lyophilization method and applied for the first time in the photodegradation of ibuprofen. The STEM images and mapping of the selected area showed that graphene oxide layers are intercalated in 2D TiO_2_ and embedded with ZnFe_2_O_4_. The obtained nanocomposite 2D/2D TiO_2_-GO-ZnFe_2_O_4_ showed a nearly anhysteretic magnetic response, together with a high initial magnetic susceptibility value, enabling effective magnetic separation. The valence band edge location estimated according to a value of the flat band edge position and bandgap energy correlates with significant inhibition of IBU degradation in the presence of superoxide radicals. Furthermore, the photodegradation tests in the presence of scavengers and the electronic spin resonance ESR analysis indicated the significant involvement of superoxide radicals and singlet oxygen in the IBU photodegradation. Based on LC–MS analysis, intermediates were determined in the photocatalytic reaction, and a pathway of photodegradation was proposed. For 2D/2D TiO_2_-GO-ZnFe_2_O_4_ composite, ibuprofen photocatalytic degradation reached 90% in only 20 min, which proved that the 2D magnetic composite is a promising photocatalyst for the degradation of active pharmaceutical ingredients under solar light. The photolysis of ibuprofen led to the formation of more toxic intermediates than the parent compound, whereas photodegradation in the presence of 2D/2D TiO_2_-GO-ZnFe_2_O_4_ composite led to non-toxic and more susceptible to biodegradation intermediates.

## Data Availability

We declared that the data and materials presented in this paper are reliable.
